# C1QC, VSIG4, and CFD as Potential Peripheral Blood Biomarkers in Atrial Fibrillation-Related Cardioembolic Stroke

**DOI:** 10.1155/2023/5199810

**Published:** 2023-01-05

**Authors:** Qian Ding, Juan Xing, Fanghui Bai, Wei Shao, Kaiqi Hou, Shoudu Zhang, Yuanzheng Hu, Baochao Zhang, Hui Zhao, Qian Xu

**Affiliations:** ^1^Henan Provincial Engineering Laboratory of Insects Bio-Reactor, Nanyang Normal University, Nanyang, China; ^2^Henan Provincial Key Laboratory of Stroke Prevention and Treatment. Nanyang central Hospital, Nanyang, China; ^3^Zhengzhou Revogene Lnc, Zhengzhou, China

## Abstract

Atrial fibrillation (AF) is a major risk factor for ischemic stroke. We aimed to identify novel potential biomarkers with diagnostic value in patients with atrial fibrillation-related cardioembolic stroke (AF-CE).Publicly available gene expression profiles related to AF, cardioembolic stroke (CE), and large artery atherosclerosis (LAA) were downloaded from the Gene Expression Omnibus (GEO). Differentially expressed genes (DEGs) were identified and then functionally annotated. The support vector machine recursive feature elimination (SVM-RFE) and least absolute shrinkage and selection operator (LASSO) regression analysis were conducted to identify potential diagnostic AF-CE biomarkers. Furthermore, the results were validated by using external data sets, and discriminability was measured by the area under the ROC curve (AUC). In order to verify the predictive results, the blood samples of 13 healthy controls, 20 patients with CE, and 20 patients with LAA stroke were acquired for RT-qPCR, and the correlation between biomarkers and clinical features was further explored. Lastly, a nomogram and the companion website were developed to predict the CE-risk rate. Three feature genes (C1QC, VSIG4, and CFD) were selected and validated in the training and the external datasets. The qRT-PCR evaluation showed that the levels of blood biomarkers (C1QC, VSIG4, and CFD) in patients with AF-CE can be used to differentiate patients with AF-CE from normal controls (*P* < 0.05) and can effectively discriminate AF-CE from LAA stroke (*P* < 0.05). Immune cell infiltration analysis revealed that three feature genes were correlated with immune system such as neutrophils. Clinical impact curve, calibration curves, ROC, and DCAs of the nomogram indicate that the nomogram had good performance. Our findings showed that C1QC, VSIG4, and CFD can potentially serve as diagnostic blood biomarkers of AF-CE; novel nomogram and the companion website can help clinicians to identify high-risk individuals, thus helping to guide treatment decisions for stroke patients.

## 1. Introduction

Ischaemic stroke accounts for the majority of stroke cases [1]. Approximately one-fifth of all ischaemic strokes are CE, and atherosclerosis causes stroke in one-fifth of cases [2]. The biochemical pathways differ between ischaemic stroke subtypes (e.g., LAA stroke versus CE). Atrial fibrillation (AF), characterized by rapid and abnormal atrial electrical activity, is the most common type of supraventricular tachyarrhythmia.

Among patients with ischemic stroke, the presence of AF is an important risk factor for ischemic stroke and for recurrent ischemic stroke, whether the type is paroxysmal AF (PAF) or permanent AF [3]. Also, antiplatelet therapy is applied to thrombotic stroke, while anticoagulant is indicated for cardioembolism caused by AF [4]. However, patients with embolic stroke of undetermined source may require long-term monitoring to detect paroxysmal AF or silent. Additionally, neither clinical characteristics nor neuroimaging findings alone can reliably classify the underlying cause of CE; in the absence of characteristic imaging features, the clinician may miss diagnosing CE strokes located at extremely unusual locations [5, 6]. Therefore, it is necessary to identify biomarkers to distinguish patients with AF-CE from normal controls and can discriminate AF-CE from other types of ischaemic stroke.

Blood-based biomarkers for differentiating stroke subtypes include but are not limited to interleukin-6 (IL-6) [7], D-dimer [8, 9], C-reactive protein (CRP) [10], and B-type natriuretic peptide (BNP) [11, 12]. In recent years, with the development of microarray technology, machine learning, and integrated bioinformatics analysis, novel disease-related genes have been identified and demonstrated to be diagnostic biological markers and treatment targets [13]. For example, in a study using group least absolute shrinkage and selector operation (LASSO) and support vector machine-recursive feature elimination (SVM-RFE), Li et al. screened seven key risk genes for Alzheimer's disease [14]. In addition, Yang et al. identified an 11-gene combination as an optimal postmenopausal osteoporosis reference biomarker by using machine learning [15]. Moreover, research has shown that the immune system plays multiple roles in stroke, and key immune cell subtypes have been increasingly recognized as diagnostic factors [16]. Up to now, there is no blood-based biomarkers for differentiating patients with AF-CE from normal controls and discriminate AF-CE from ischaemic stroke subtypes though machine learning.

In this study, following performing a comprehensive analysis of coexpressed DEGs of persistent AF and CE, two algorithms, LASSO and SVM-RFE, were used to select diagnostic markers for AF-CE. Then, the predictive value of biomarkers was estimated in the training set, verification set, and test clinical samples, in order to evaluate whether the levels of blood biomarkers in patients with AF-CE can be used to differentiate patients with AF-CE from normal controls and can effectively discriminate AF-CE from ischaemic stroke subtypes (LAA stroke).

## 2. Materials and Methods

### 2.1. Data Information and Processing

The GSE58294 [17], GSE41177 [18], GSE14975 [19], GSE115574 [20], and GSE20129 [21] datasets were obtained from the GEO database (http://www.ncbi.nlm.nih.gov/geo) (Supplementary Table S1). The GSE58294 (GPL570) dataset includes 23 blood samples from patients with CE and 23 controls without symptomatic vascular diseases and the results of analyses at three time points (<3 h, 5 h, and 24 h) following the stroke event. The GSE41177 dataset contains 19 samples, including 16 left atria junctions of patients with AF versus 3 controls with sinus rhythm (SR). The GSE115574 dataset contains 29 samples, including 14 patients with AF and 15 with SR. The GSE14975 dataset includes 5 patients with AF and 5 with SR. The GSE20129 dataset comprises 57 samples with atherosclerosis and 78 normal controls. The GSE58294 and GSE41177 datasets were merged as the training set, and batch effects were directly adjusted using the combat function in SVA. The GSE14975 and GSE115574 datasets were merged as the verification set, and the GSE20129 dataset was utilized as the reverse validation set. All the datasets were subjected to standardized data preprocessing.

### 2.2. Identifying DEGs

By using GEO2R (http://www.ncbi.nlm.nih.gov/geo/geo2r), a tool provided by the GEO database that relies on the R package “limma”, CE-related DEGs were screened from GSE58294 (GPL570) dataset, at each of the three time points (<3 h, 5 h, and 24 h) separately compared to the controls. AF-related DEGs were screened from the GSE41177 dataset. Meanwhile, AF-CE-related DEGs were also filtrated from the merged GSE58294 and GSE41177 training set. Screening criteria for DEGs were an adjusted *P* value < 0.05 and |log2 − fold − change (FC)| > 1. The “ggplot2” package was used to create a volcano plot of DEGs. Overlapping DEGs were extracted and visualized for further analysis by using the Venn diagram web tool (http://bioinformatics.psb.ugent.be/webtools/Venn/).

### 2.3. Functional Enrichment Analysis

To understand the function of DEGs in AF-CE patients, bioinformatic analyses for clustering 316 AF-CE-related DEGs were performed using Metascape and DO analyses. The Metascape data platform (http://metascape.org) [22] was used for functional enrichment analysis, and the results were visualized using biological online tools. The human disease ontology (DO) [23] (http://www.disease-ontology.org) is a community-driven standard-based ontology that is focused on annotating genes based on human disease, which was performed using the DOSE packages and “clusterProfiler” in R.

### 2.4. Diagnostic Biomarker Screening

Artificial intelligence (AI) is achieved by using machine learning to analyse existing data and obtain rules or models that are then used to predict unknown variables. Here, two algorithms, the dimension reduction approach LASSO and SVM-RFE, were used. Compared to regression analysis, LASSO algorithms were performed with a turning/penalty parameter and were better at evaluating high-dimensional data through the “glmnet” package [24]. SVM-RFE algorithms [25] were superior to linear discriminant analysis and mean square error, which can be used to select relevant variables in place of linear discriminant analysis, remove redundant variables by deleting SVM-generated eigenvectors and cross-validate tenfold, and were used to select candidate genes through the “glmnet” package. We screened the overlapping genes for further analysis.

### 2.5. Diagnostic Value of Feature Biomarkers in AF-CE

Next, we estimated the predictive value of biomarkers by quantifying their sensitivity and specificity using receiver operating characteristic (ROC) curve analysis and measurement of the AUC. Based on the ROC curves, the optimal cut-off value was calculated for the predictive value of the feature biomarkers in the training set and further validated in the verification and reverse verification sets.

### 2.6. Patients and Variables

The data of the study population were collected from August 2021 to April 2022. A total of 40 patients, 20 patients with LAA stroke and 20 patients with CE, were enrolled consecutively from Nanyang Central Hospital (Nanyang, Henan, China).

Disease diagnosis was based on a history of illness, clinical performance, auxiliary examination, and case notes by at least two specialized expert neurologists. Stroke subtyping followed the *TOAST* (Trial of Org 10172 in acute stroke treatment) classification [26]. The inclusion and exclusion criteria of CE were as follows: (1) cerebral embolism caused by obstruction of blood vessels in the brain after they detach from their cardiogenic emboli and (2) at least one cardiac-derived risk factor. The inclusion and exclusion criteria of LAA were as follows: (1) imaging showed that common carotid artery, anterior and posterior cerebral artery and vertebrobasilar artery occlusion or stenosis, was >50%; (2) lesions were caused by atherosclerosis; (3) audible murmur on neck auscultation; (4) imaging showed a lesion diameter > 1.5 cm; (5) cardioembolic stroke and stroke from other causes were excluded. Thirteen age- and sex-matched healthy volunteers were recruited as healthy controls. The volunteers had no history of neurologic events (cerebrovascular stroke or transient ischaemic attack). All the experiments were carried out in accordance with the Nanyang Central Hospital Ethics Committee's guidelines and regulations. Institutional ethics committee approval was obtained for this study, and informed consent was obtained from all patients or their relatives.

### 2.7. Total RNA Extraction and Quantitative Real-Time PCR Expression Analysis

Peripheral blood samples were immediately preserved in blood RNA storage tubes (BioTeke Corporation. Beijing, China) for all RNAs isolated from peripheral blood samples. cDNA was synthesized from total RNA by using an M5 Super plus qPCR RT kit with gDNA (Mei5 Biotechnology, Co., Ltd., Beijing, China). qRT-PCR was performed using the CFX96TM Real-Time System (Bio-Rad, USA), and a comparative quantification was conducted. GAPDH served as the internal reference. The sequences of the primers used were as follows: C1QC-F: CATCCTTGCCTAGACCATTC, C1QC-R: GTACCAGAAGGCATTGGTTA, VSIG4-F: AGAGAGTGTAACAGGACCTT, VSIG4-R: GTCACGTAGAAAGATGGTGA, CFD-F: CTCCAAGCGCCTGTACGAC, CFD-R: CAGTGTGGCCTTCTCCGAC.

### 2.8. Discovery of Immune Cell Subtypes

AF-CE samples were analysed using the CIBERSORT algorithm [27] to determine the immune cell infiltration. For each microarray experiment, the putative proportion of immune cells as defined by 22 sorted immune cell subtypes (LM22) was calculated by using CIBERSORT (http://cibersort.stanford.edu/). Then, the violin plots were created using the R package “vioplot” with standard parameters to visualize and analyse differences in immune cell infiltration between AF-CE samples at <3, 5, and 24 hours samples and control samples.

### 2.9. Nomogram and Online Prediction Tool

A nomogram was generated using the nomogram function in the R library, which is part of the R programming language. Calibration curves were plotted to assess the performance and internal validity of the nomogram with the development cohort. Using the DecisionCurve package in R, decision and clinical impact curves were generated. An online tool for predicting risk was written in html, css, JavaScript, and jQuery language using web-based software, which can be accessed at https://www.origingenetic.com/CardiogenicStroke. With input values of predictors for potential AF-CE patients, the online calculator immediately returns predicted morbidity based on the nomogram function constructed from the expression values of candidate diagnostic genes.

### 2.10. Statistical Analysis

All statistical analyses in this study were conducted using GraphPad Prism 8.0 (https://www.graphpad.com/scientific-software/prism/) and R (version 4.1.3). The correlation between diagnostic gene expression levels and clinical factors was determined using unpaired Student's *t* tests for continuous variables and Fisher's exact tests for categorical variables. This regression was carried out using the R package glmnet to perform the LASSO test, and we trained the SVM model with the help of R package e107. To determine the diagnostic power and accuracy, we applied ROC curve analysis. For all analyses, a two-sided *P* < 0.05 was considered to indicate statistical significance.

## 3. Results

### 3.1. Identification of DEGs

**Figure 1:**
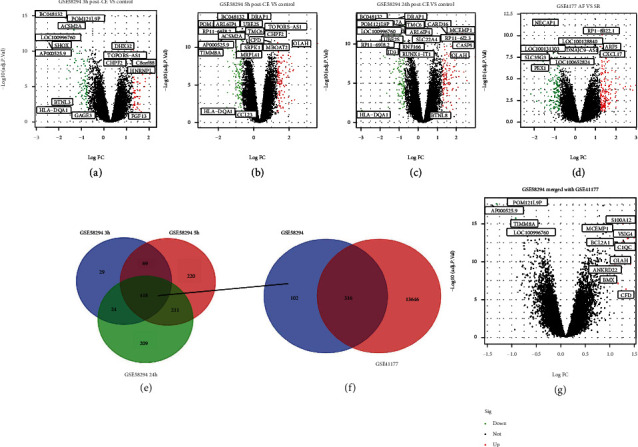
DEGs representation by volcano plot diagrams and Venn diagram.(a–c) Identification of DEGs separately in cardioembolic stroke (CE) obtained from patients with CE at each of the three time points ≤ 3 h (a), 5 h (b), and 24 h (c) following the stroke event compared to controls in GSE58294. (d) DEGs from patients with AF compared to SR in GSE41177. (e) Venn diagrams representing the number of overlapping DEGs among the patients with CE at the three time points following the stroke event compared to controls in GSE58294. (f) Venn diagrams representing the number of overlapping DEGs between CE (GSE58294) and AF (GSE41177) by GEO2R. (g) DEGs obtained from training set (GSE58294 merged with GSE41177).

In the GSE58294 (GPL570) dataset related to CE, DEGs screening separately at each of the three time points (<3 h (Figure 1(a)), 5 h (Figure 1(b)), and 24 h (Figure 1(c)) after stroke compared to the control. Meanwhile, a total of 13962 AF-related DEGs were obtained from GSE41177 (Figure 1(d)). Based on the DEGs for different time points in the GSE58294 dataset separately, we found that the intersection of the results provided 418 DEGs (Figure 1(e)). Furthermore, 316 AF-CE genes were obtained by intersecting with these DEGs (Figure 1(f)). In addition, the GSE58294 and GSE41177 datasets were merged as the training set. Using the limma package after removing the batch effects, thirteen AF-CE related DEGs genes (AP000525.9, POM121L9P, TIMM8A, MCEMP1, C1QC, LOC100996760, BCL2A1, S100A12, VSIG4, OLAH, ANKRD22, BMX, and CFD) were obtained from the merged training set, including 9 upregulated genes and 4 downregulated genes (Figure 1(g)).

### 3.2. Functional Correlation Analysis

**Figure 2:**
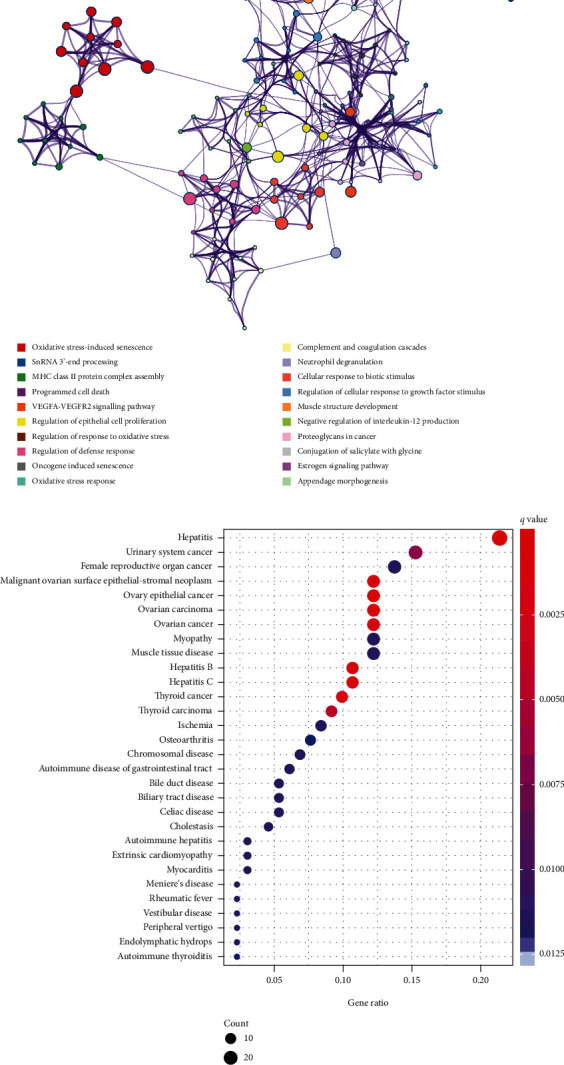
Functional enrichment analysis of the AF-CE-related DEGs. (a) The enrichment analysis of 316 AF-CE-related DEGs was performed using the Metascape online tool. (b) The DO enrichment analysis on 316 AF-CE-related DEGs.

Then, the Metascape online tool was used to functionally annotate the 316 AF-CE-related DEGs. The results revealed that AF-CE-related DEGs were markedly enriched in oxidative stress-induced senescence, oxidative stress response, regulation of response to oxidative stress, programmed cell death, regulation of epithelial cell proliferation, VEGFA-VEGFR2 signalling pathway, complement and coagulation cascades, oestrogen signalling pathway, snRNA 3′-end processing, MHC class II protein complex assembly, regulation of cellular response to growth factor stimulus, muscle structure development, negative regulation of interleukin-12 production, proteoglycans in cancer, conjugation of salicylate with glycine, and appendage morphogenesis (Figure 2(a)). The DO enrichment analysis results showed that AF-CE-related DEGs were mainly associated with immune-mediated inflammatory diseases (hepatitis, hepatitis B, and hepatitis C) and female reproductive system diseases (female reproductive organ cancer, ovarian epithelial cancer, malignant ovarian surface epithelial-stromal neoplasm, ovarian carcinoma, and urinary system cancer) (Figure 2(b)).

### 3.3. Selection of Diagnostic Markers via LASSO and SVM-RFE Algorithms

**Figure 3:**
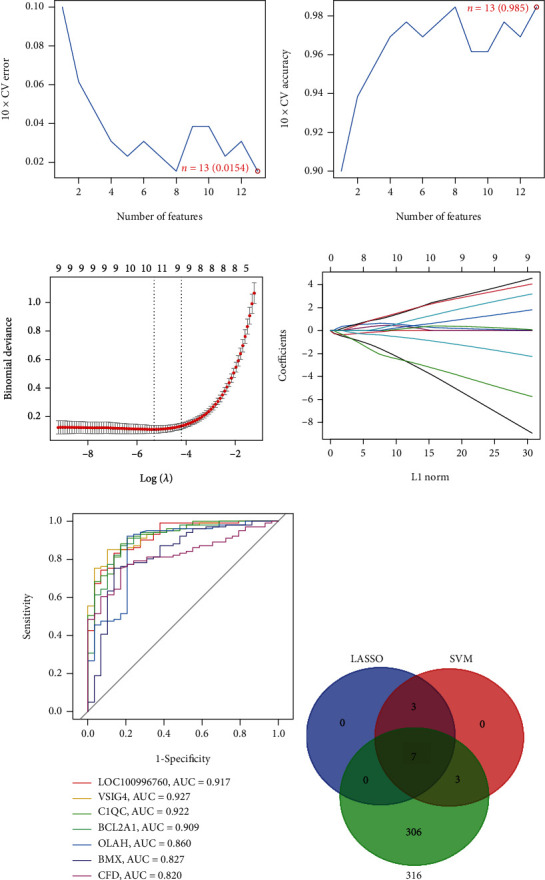
Screening of diagnostic genes. (a) The 10x cross-validation (CV) error curve of the relationship between the number of genes used for fitting and SVM-RFE model error via the SVM-RFE algorithm. (b) The 10x CV accuracy curve of the relationship between the number of genes used for fitting and SVM-RFE model accuracy via the SVM-RFE algorithm. (c) The partial likelihood deviation curve of the minimum number of signature genes. (d) The 10x CV for tuning parameter selection in the LASSO model. Each curve corresponds to a single gene. (e) Venn diagram representing the number of diagnostic markers extracted from LASSO and SVM-RFE algorithms overlapping 316 AF-CE-related DEGs. (f) ROC curve to verify the diagnostic efficacy of diagnostic genes. CV: cross-validation.

Next, two distinct algorithms (LASSO and SVM-RFE) were utilized for selecting feature genes screened from the combined (GSE58294 and GSE41177) training set. For the SVM-RFE algorithm, the results showed that the classifier produced the minimum error when the feature number was 13, containing AP000525.9, POM121L9P, TIMM8A, MCEMP1, C1QC, LOC100996760, BCL2A1, S100A12, VSIG4, OLAH, ANKRD22, BMX, and CFD (Figures 3(a) and 3(b)). For the LASSO algorithm, following tenfold cross validation, a set of 10 genes was selected, including AP000525.9, TIMM8A, S100A12, LOC100996760, VSIG4, C1QC, BCL2A1, OLAH, BMX, and CFD (Figures 3(c) and 3(d)). Overall, 7 feature genes (LOC100996760, VSIG4, C1QC, BCL2A1, OLAH, BMX, and CFD) shared between the LASSO and SVM-RFE algorithms and GEO2R-screened DEGs as diagnostic markers for AF-CE patients were finally selected for further analysis (Figure 3(e)). Notably, the AUC values of ROC analysis for the 7 feature genes were all greater than 0.8, which suggested that these 7 genes might serve as diagnostic markers for AF-CE patients (Figure 3(f)).

**Figure 4:**
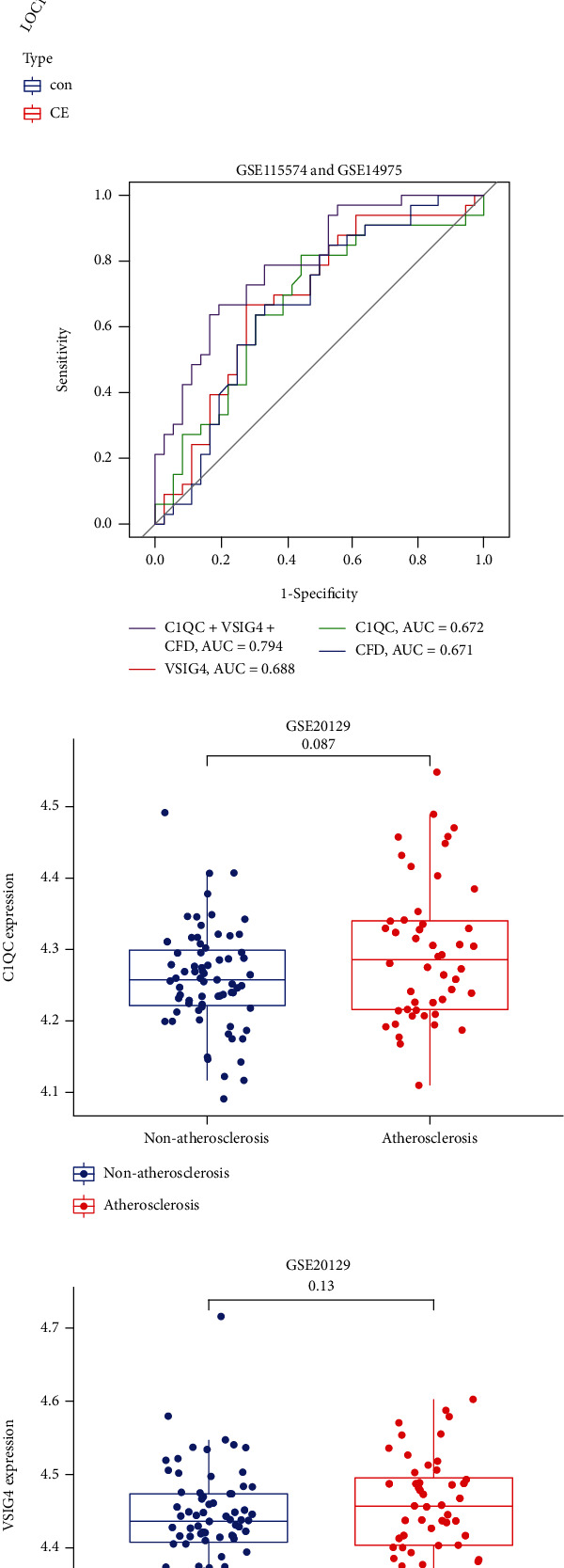
Validation of the feature biomarkers. Validation of the expression of the feature biomarkers by (a) heatmap and (b) boxplot in the validation set (GSE115574 and GSE14975 pooled datasets). ^∗^*P* < 0.05, ^∗∗^*P* < 0.01. (c) ROC curve to verify the diagnostic efficacy of the feature biomarkers in the validation set. (d–f) validation of the expression of the feature biomarkers (d)C1QC, (e)VSIG4, and (f) CFD in the reverse verification set (GSE20129).

Moreover, to further validate the reliability and reproducibility of the seven candidate diagnostic genes, we merged two datasets (GSE115574 and GSE14975) as a validation set. The results showed that CFD (*P* < 0.05), VSIG4 (*P* < 0.01), and C1QC (*P* < 0.05) were differentially expressed between AF and SR (Figures 4(a) and 4(b)). Then, a powerful discrimination ability was confirmed in the ROC analysis. As shown in Figure 4(c), there was an AUC of 0.672 in C1QC, an AUC of 0.688 in VSIG4, an AUC of 0.671 in CFD, and an AUC of 0.794 in the combined three-genes (C1QC+VSIG4+CFD) model. The AUC of model was higher than those of the individual genes, which had a higher diagnostic value. However, there was no significant difference between the expression of C1QC, VSIG4 and CFD in the atherosclerosis dataset GSE20129 (Figures 4(d)–4(f)). From the results above, the feature biomarkers VSIG4, C1QC, and CFD were determined to have high diagnostic accuracy.

### 3.4. Pathway Analysis of the Feature Biomarkers

**Figure 5:**
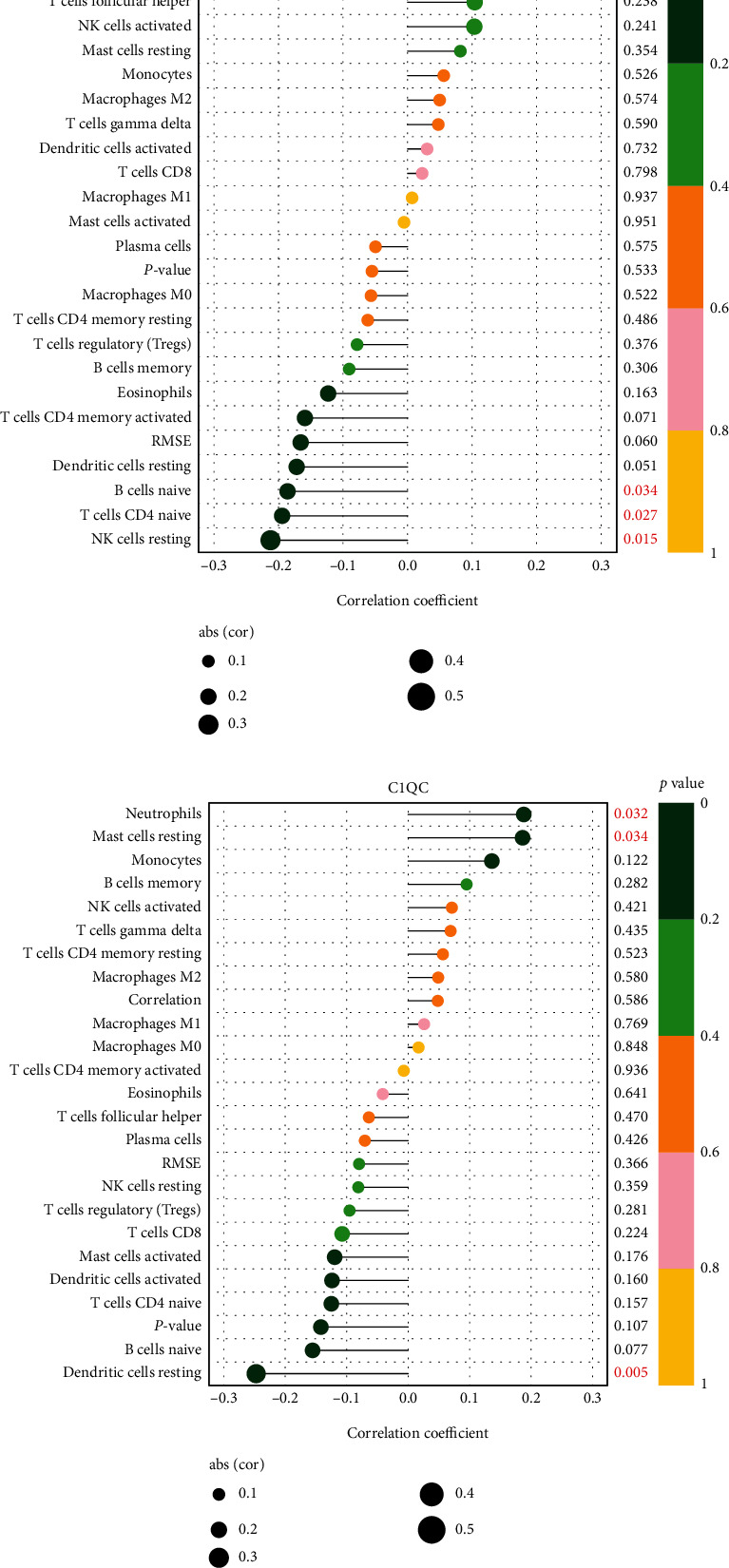
Function enrichment analysis of candidate diagnostic biomarkers. (a) Enrichment analysis of C1QC, CFD, and VSIG4. (b) Correlation between CFD expression and infiltrating immune cells. (c) Correlation between C1QC expression and infiltrating immune cells. (d) Correlation between VSIG4 expression and infiltrating immune cells.

To define the biological relevance of VSIG4, C1QC, and CFD, we performed enrichment analysis from the PathCards database (https://pathcards.genecards.org/). The results showed that the candidate diagnostic genes were mainly enriched in the immune response lectin-induced complement pathway, formation of fibrin clots (clotting cascade), creation of C4 and C2 activators, innate immune system, complement pathway, complement and coagulation cascades, response to elevated platelet cytosolic Ca^2+^, and adipogenesis (Figure 5(a)). We propose that VSIG4, C1QC, and CFD may be associated with a regulated cellular immune response of AF-CE which in turn influences AF-CE risk.

### 3.5. Immune Cell Infiltration

We then calculated the difference in the distribution of immune cell infiltration in the GSE58294 dataset between patient samples, <3, 5, and 24 h after stroke and control samples. When compared with the control group, neutrophils (*P* < 0.05) were higher in the AF-CE group at <3, 5, and 24 h after stroke; M0 (*P* < 0.05) and M2 macrophages (*P* < 0.05) were higher in the AF-CE group at 5 h after stroke; T cells gamma delta (*P* < 0.05) and M2 macrophages (*P* < 0.05) were higher in the AF-CE group at 24 h after stroke; resting dendritic cells (*P* < 0.05) were significantly lower at 3 h and 5 h after stroke. In addition, naive CD4 T cells (*P* < 0.05) were lower at 3 h after stroke, eosinophils (*P* < 0.05) were lower at 5 h, and CD8 T cells (*P* < 0.05) and resting NK cells (*P* < 0.05) were lower at 24 h (Supplement Figure S1).

For the feature biomarkers (VSIG4, C1QC, and CFD), we found that CFD was positively correlated with neutrophils (*P* = 0.007) but negatively associated with naive B cells (*P* = 0.034), naive CD4 T cells (*P* = 0.027), and resting NK cells (*P* = 0.015, Figure 5(b)). C1QC was positively correlated with neutrophils (*P* = 0.032) and resting mast cells (*P* = 0.034) but negatively correlated with resting dendritic cells (*P* = 0.05, Figure 5(c)). Moreover, VSIG4 was positively associated with neutrophils (*P* < 0.001) and negatively correlated with T follicular helper cells (*P* = 0.039), resting dendritic cells (*P* = 0.010), and eosinophils (*P* = 0.001, Figure 5(d)). These findings agree with the results of the pathway analysis.

### 3.6. Expression of Diagnostic Genes in Clinical Samples

**Figure 6:**
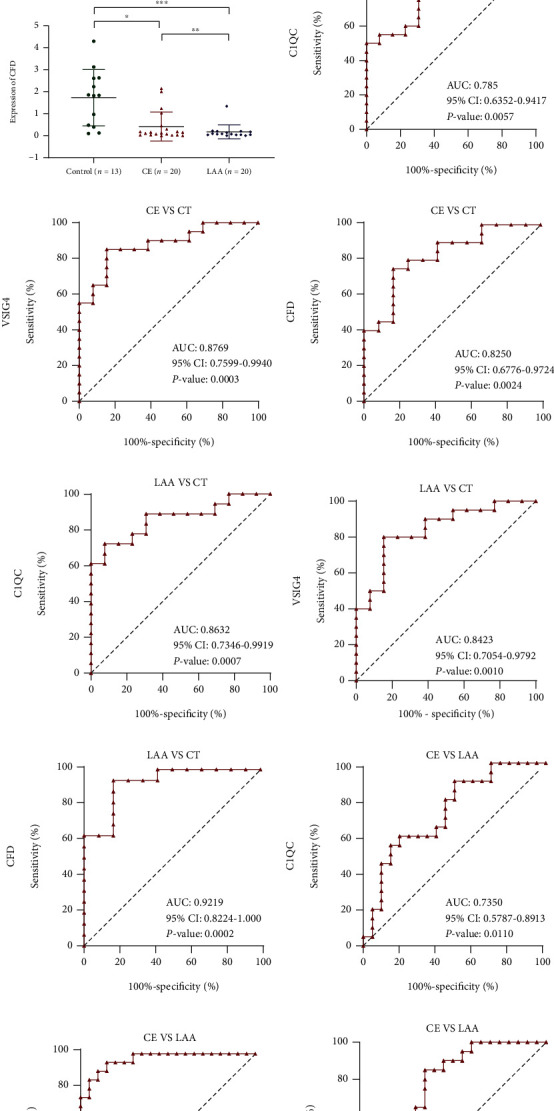
Diagnostic value of the three diagnostic genes in AF-CE. The expression levels of (a) C1QC, (b) VSIG4, and (c) CFD levels were analysed by RT-qPCR. (d–l) ROC curves of C1QC, VSIG4, and CFD.^∗^*P* < 0.05, ^∗∗^*P* < 0.01, ^∗∗∗^*P* < 0.001. Horizontal lines represent median levels and interquartile ranges. CT: control; CE: cardioembolic stroke; LAA: large-artery atherosclerosis stroke.

To further confirm our findings, a total of 53 clinical blood samples (20 patients with LAA, 20 patients with CE, and 13 controls) from patients were collected, and qRT–PCR analyses were performed to measure the expression of C1QC, VSIG4, and CFD. Clinical information of the patient samples is summarized in Supplement Table S2. As shown in Figures 6(a)–6(c), the expression of C1QC (*P* < 0.001) and VSIG4 (*P* < 0.001) in the CE and LAA groups was significantly increased, but the expression of CFD (*P* < 0.001) was decreased in the CE and LAA groups compared with the healthy control group, which was supported by the bioinformatics analysis.

### 3.7. Assessment of C1QC, VSIG4, and CFD as Potential AF-CE Biomarkers

To evaluate the diagnostic accuracy of a candidate diagnostic gene, an ROC curve was constructed. When comparing controls with CE patients, the AUC of C1QC was 0.7885 (95% confidence interval (CI): 0.6352–0.9417; *P* = 0.0057) (Figure 6(d)), the AUC of VSIG4 was 0.8769 (95% CI: 0.7599–0.9940; *P* = 0.0003) (Figure 6(e)), and the AUC of CFD was 0.8250 (95% CI: 0.6776–0.9724; *P* = 0.0024) (Figure 6(f)). In addition, when comparing controls and LAA patients, the AUC of C1QC was 0.8632 (95% CI: 0.7346–0.9919, *P* = 0.0007) (Figure 6(g)), the AUC of VSIG4 was 0.8423 (95% CI: 0.7054–0.9792, *P* = 0.0010) (Figure 6(h)), and the AUC of CFD was 0.9219 (95% CI: 0.8224–1.000, *P* = 0.0002) (Figure 6(i)). In addition, when comparing CE patients with LAA patients, the AUC of C1QC was 0.7350 (95% CI: 0.5787–0.8913, *P* = 0.0110) (Figure 6(j)), the AUC of VSIG4 was 0.9675 (95% CI: 0.9226–1.000, *P* < 0.0001) (Figure 6(k)), and the AUC of CFD was 0.7575 (95% CI: 0.6060–0.9090, *P* = 0.0053) (Figure 6(l)). These results firmly show that C1QC, VSIG4, and CFD expression offers great value in differentiating between controls and CE and LAA patients and is specific for the two stroke subtypes analysed. As such, our findings suggest that C1QC, VSIG4, and CFD may represent a diagnostic biomarker for AF-CE.

### 3.8. Construction and Evaluation of the AF-CE Diagnostic Nomogram and Online Prediction Tool

**Figure 7:**
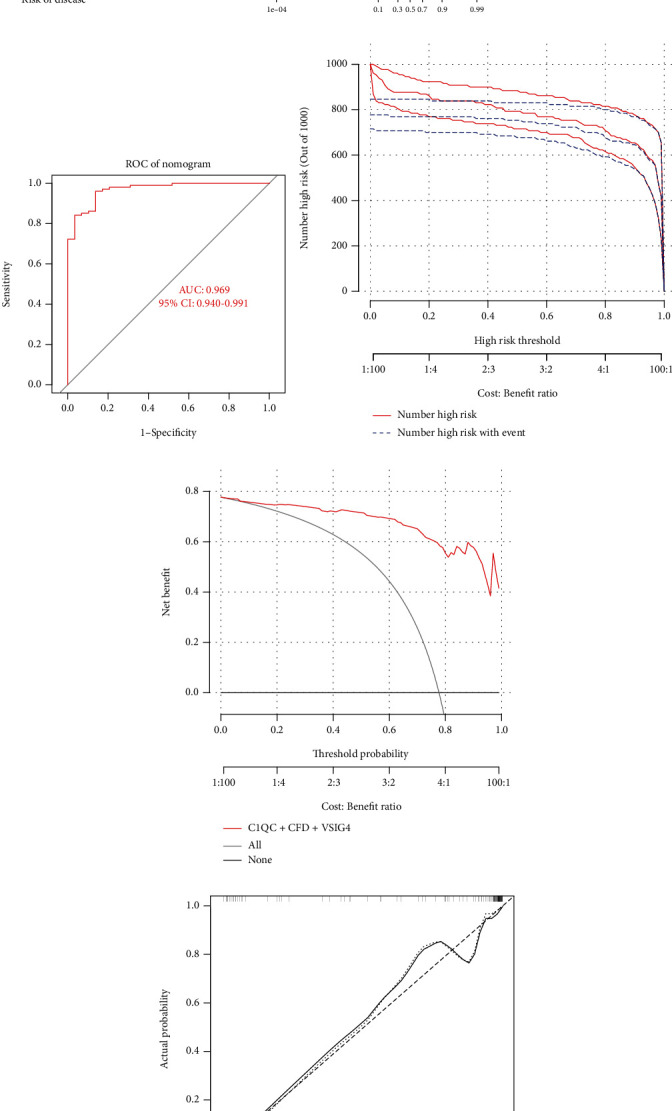
Nomogram predicting the risk of AF-CE. (a) Nomogram with three peripheral blood biomarkers in AF-CE, (b) ROC curve, (c) clinical impact curve, (d) decision curve, and (e) calibration curve.

A nomogram was constructed to diagnose AF-CE based on the 3 diagnostic genes (C1QC, CFD, and VSIG4) by using the “RMS” package((Figure 7(a)). Then, to evaluate the clinical effect of the nomogram model more intuitively, a clinical impact curve was calculated based on the curve generated by decision curve analysis (DCA). The high-risk curve represented in red was very close to the true positive patient curve represented in blue. There was good discrimination efficacy with the AF-CE nomogram, with an area under the curve (AUC) of 0.969 (95% CI: 0.940-0.991) (Figure 7(b)). This indicated that the nomogram model was capable and acceptable for predicting discrimination accurately (Figure 7(c)). DCA showed that the C1QC+CFD+VSIG4 curve was much higher than the grey line, which explains the high accuracy of the nomogram model (Figure 7(d)). In addition, a calibration curve was constructed to determine the predictive ability of the nomogram model. According to the calibration curve, there was little difference between the actual and predicted risk of AF-CE, indicating a high degree of accuracy in predicting AF-CE (Figure 7(e)). Collectively, the evidence supports the ability of the new nomogram to assess and predict the risk prediction of AF-CE. Furthermore, for clinical utility, we developed an online prediction tool (https://www.origingenetic.com/CardiogenicStroke) to predict the risk of AF-CE based on the constructed nomogram. We entered the expression levels of the candidate diagnostic genes (C1QC, CFD, and VSIG4) into the online prediction tool. As expected, the results of the clinical validation showed that all AF-CE patient test sample scores were nearly 100%, whereas healthy controls were nearly 0%, which implies that the prediction software has high delineation accuracy.

### 3.9. Correlations between Clinicopathological Parameters and Candidate Disease Biomarkers

**Figure 8:**
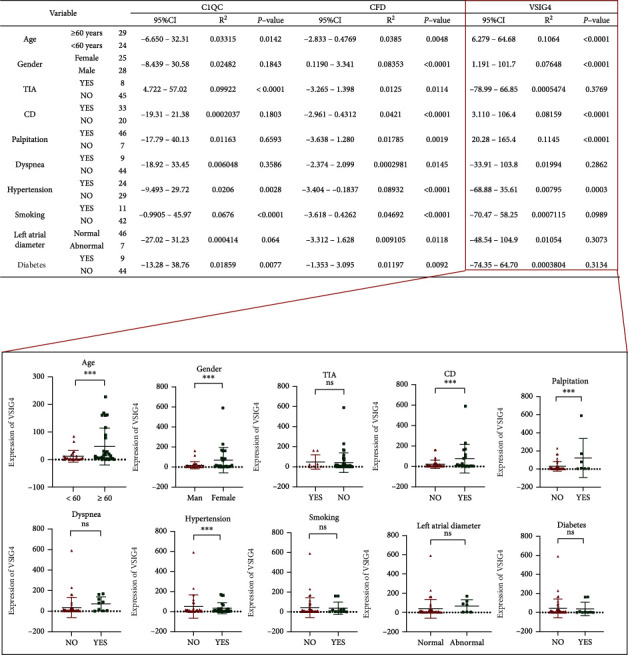
Correlation of C1QC, CFD, and VSIG4 with clinicopathological characteristics. TIA: transient ischemic attack; CD: cardiovascular disease. ^∗^*P* < 0.05, ^∗∗^*P* < 0.01, ^∗∗∗^*P* < 0.001.

To clarify the roles of candidate disease characteristic biomarkers in the development of AF-CE, the connections between the expression of the three candidate genes (C1QC, CFD, and VSIG4) and clinical pathological features (including age, sex, TIA, cardiogenic diseases, palpitation, dyspnoea, hypertension, smoking, left atrial diameter, and diabetes) in AF-CE patients were analysed. As shown in Figure 8, in comparing the general data, the expression of C1QC was positively correlated with age (>60 years) (*P* < 0.05), history of diabetes (*P* < 0.05), history of hypertension (*P* < 0.05), and current or recent smoking (*P* < 0.05), while no interrelation was discovered between C1QC expression and the other clinicopathological parameters of the patients (*P* > 0.05). Interestingly, for CFD expression, there were significant differences in all of the above clinical pathological features. For instance, compared with no history of diabetes, a history of diabetes was associated with a lower expression of CFD (*P* < 0.05); compared with males, females had a lower expression of CFD (*P* < 0.05); compared with a no history of hypertension, a history of hypertension was associated with a lower expression of CFD (*P* < 0.05). In addition, VSIG4 expression was remarkably higher among patients who had a history of hypertension (*P* < 0.05), cardiogenic diseases (*P* < 0.05), and palpitations (*P* < 0.05); smokers (*P* < 0.05); patients aged >60 years (*P* < 0.05); females (*P* < 0.05).

## 4. Discussion

Cardiogenic stroke is one of the most lethal types of ischaemic stroke and is predominantly caused by a cardiogenic embolus (or emboli) breaking off and cleaving to a corresponding cerebral artery [28]. Ischaemic stroke is caused by multiple factors, including environmental and lifestyle causes, which can make phenotypic assortment difficult [29]. AF is thought to be the most common form of arrhythmia and the leading cause of cardioembolic stroke [30]. In general, AF includes paroxysmal atrial fibrillation (PAF) and persistent atrial fibrillation (PeAF) [31]. Moreover, previous studies have demonstrated that patients with CE or strokes with AF appeared to be at greater risk of haemorrhagic transformation [32]. In the diagnosis of CE, echocardiography (ECG) is one of the most important examinations [33]. However, AF is often paroxysmal and asymptomatic; therefore, widely used rest ECG monitoring could not detect all paroxysmal AF [34]. In this study, our goal was to identify mRNAs that might serve as diagnostic biomarkers for AF-CE.

The major purpose for subtyping cardiogenic cerebral embolism patients is to develop a better therapeutic decision-making process. Existing methods are outdated, time consuming (hours to days), complex, and expensive. Currently, blood biomarkers may be used to predict and diagnose ischaemic stroke [35], including B-type natriuretic peptide, interleukin-6, D-dimer, total cholesterol, interleukin-1*β*, and high-density lipoprotein. In recent years, mRNAs and miRNAs have been used as promising biomarkers in cardiovascular and stroke diseases. For example, during TIMP4 inhibition, miR-146b-5p promotes atrial fibrosis in patients with AF [36]. In particular, serum miR-125a-5p, miR-125b-5p, and miR-433-5p are potential biomarkers for distinguishing between peripheral vertigo and posterior circulation stroke [37]. With the development of artificial intelligence, machine learning has recently become utilized in the screening, diagnosis, and prognosis of disease (e.g., the prediction of heart failure [38], the detection of hepatocellular carcinoma [39], the diagnosis of diabetic retinopathy [40], and the prediction of hip fractures [41]). Regrettably, in the absence of early diagnosis and effective therapies, the prognosis of AF-CE is currently unsatisfactory. Therefore, we explored for the first time the specific blood-based biomarkers that could enable the rapid diagnosis of patients with AF-CE, which may serve to support treatment planning and secondary prevention programs, by using machine learning.

According to our findings, the identified DEGs were enriched in oxidative stress-induced senescence, programmed cell death, the VEGFA-VEGFR2 signalling pathway, and the complement and coagulation cascades signalling pathway. Moskowitz et al. and Maida et al. reported that excitotoxicity, oxidative stress, and inflammation were significant risk factors for brain injury caused by ischaemia [42, 43]. Jiang et al. found that oxidative stress and inflammation were associated with the pathogenesis of cardio-cerebrovascular disease (CCVD) and were closely associated with senescent vascular endothelial cells [44]. In addition, stroke triggers an inflammatory response, which may exacerbate brain injury. Li et al. confirmed that VEGFA-induced VEGFR2 homodimerization in hypoxia-induced VEGFA/VEGFR2 signalling predicts the treatment outcome for LAA stroke patients [45]. Additionally, sex has been well established as a known risk factor for stroke [46]. The primary female sex hormone, oestrogen, influences cardiovascular functions through the ER*α* receptor [47]. Interestingly, the DEGs were also enriched in the oestrogen signalling pathway. Tang et al. reported that the increased risk of ischaemic stroke is in part attributed to hypercoagulability induced by oestrogen [48]. There is significant evidence suggesting that oestrogen modulates cardiovascular physiology [49]. According to this evidence, the findings of our study are extremely accurate and acceptable.

In this study, three diagnostic markers were identified based on the results from two machine learning algorithms. Previous studies have found that C1QC encodes a major constituent of the human complement subcomponent C1q, which is widely expressed in various types of human malignancies and diseases [50]. It has also been shown that C1QC is associated with the complement system and increased in the inflammatory response [51]. VSIG4 encodes an M*φ*-associated complement receptor. Previous studies have found that VSIG4 is a potential biomarker of enhanced ageing in murine adipose tissue [52]. Recently, C1QC and VSIG4 were identified as potential crucial genes associated with the maintenance of cognitively normal brain ageing via bioinformatic analysis by Xu et al. [53]. The CFD gene encodes a C3-convertase that activates and amplifies alternative complement pathways. CFD is essential not only for innate immunity but also for other physiological processes [54]. Our findings showed that three diagnostic genes were mainly enriched in the immune response lectin-induced complement pathway and complement pathway. Immune reactions can be considered a useful signal for the early detection of CE as an early pathological change in this disease. A pathogen is first attacked by the complement system [55]. Tomonobu et al. [56] reported that dysregulation of the complement cascade may lead to a variety of chronic diseases, which may contribute to the development of thrombosis, systemic inflammation, and autoimmune diseases.

We then analysed the expression levels of the 3 diagnostic markers in blood samples by RT–qPCR which showed that the levels of 3 blood biomarkers in patients with AF-CE can be used to differentiate patients with AF-CE from normal controls and can effectively discriminate AF-CE from LAA stroke. Specifically compared with the control group, C1QC and VSIG4 in AF-CE patients were significantly upregulated, and the expression of CFD was significantly downregulated. The expression of CFD was significantly higher in the control group than in the test group, suggesting that CFD may be a protective gene in the development of disease. Decreased expression of CFD also implies dysregulation of the complement system, a precursor to thrombosis that predicts the risks of AF-CE. Of note, we validated our results using clinical samples, increasing the accuracy of the results. A complex and substantial influence of sex and age can be seen in the risk, outcome, and pathophysiology of ischaemic stroke [57]. Past studies have shown that stroke is an illness of ageing—most strokes occur in people >60 years old. The mortality rate and poorer quality of life of older stroke patients are higher than those of younger patients [58]. Many aspects of ischaemic stroke, including stroke risk, outcome, and treatment are influenced by sex [59]. Stroke rates continue to increase in women after middle age, and older women (age > 85 years) have higher rates of stroke than men of the same age, which is consistent with our study [60]. Previous studies have shown that a high risk of early stroke and poorer long-term survival are often associated with untreated TIA [61]. Our results show that the high expression of C1QC and VSIG4 was positively correlated with age > 60 years old, sex, history of hypertension, diabetes, smoking, and transient ischaemic attack, and the low expression of CFD was inversely related to age > 60 years old, female sex, history of hypertension, diabetes, smoking, and transient ischaemic attack. In addition, the diagnostic values of these diagnostic genes were analysed using ROC curve analysis. Each of the diagnostic genes had a reliable diagnostic value, and they all exhibited significant specificity and sensitivity. In conclusion, the above evidence demonstrates that C1QC, VSIG4, and CFD can be used as independent factors and diagnostic criteria for AF-CE.

Finally, based on these variables, we established a nomogram model to help clinicians predict the risk of AF-CE. The high concordance for the calibration curve indicates that our nomogram's discriminative ability and universal clinical applicability were validated. Then, an online tool based on the nomogram model was created. According to the prediction tool, patients with disease risk are expressed as a percentage; values closer to 100% indicate a greater likelihood of AF-CE. Online prediction tool development has largely facilitated the application of nomograms in the clinic, thereby better assisting clinicians in the evaluation and selection of treatment options for their patients. The results of the clinical validation also showed the high delineation accuracy of this online prediction tool.

However, this study had some limitations that should be noted. First, this experiment involved a small sample size and limited clinical characteristics for patient inclusion, which may lead to a bias in the experimental results. Second, several datasets with different numbers of controls impair the interpretation of the results. In the next step, we will continue to collect cases to conduct multicentre, large-sample research to confirm our findings. In addition, future research should also explore the pathogenesis of AF-CE and the pathways related to C1QC, VSIG4, and CFD in the disease in vivo and in vitro. Despite the shortcomings of this study, C1QC, VSIG4, and CFD will certainly play a remarkable role in the diagnosis and treatment of AF-CE, thus providing new methods and targets for the study of this disease.

## 5. Conclusions

In conclusion, C1QC, VSIG4, and CFD should be considered novel diagnostic biomarkers for AF-CE. This was further supported by bioinformatic analysis and experiments. Moreover, we found that the three candidate diagnostic genes were mainly enriched in the immune system and complement pathways, which could form the basis for further research. Finally, we constructed a nomogram and a suitable and convenient online tool (https://www.origingenetic.com/CardiogenicStroke) with three peripheral blood biomarkers to assist clinicians in predicting the risks for estimating the status of patients with AF-CE to make a better plan for the treatment of stroke patients.

## Data Availability

The datasets (GSE58294, GSE41177, GSE14975, GSE115574, and GSE20129) for this study can be found in GEO (https://www.ncbi.nlm.nih.gov/geo/).

## Abbreviations

AF: Atrial fibrillation
AF-CE: Atrial fibrillation-related cardioembolic stroke
CE: Cardioembolic stroke
LAA: Large artery atherosclerosis
GEO: Gene Expression Omnibus
DEG: Differentially expressed genes
SVM-RFE: Support vector machine recursive feature elimination
LASSO: Least absolute shrinkage and selection operator
AUC: Area under the ROC curve
PAF: Paroxysmal AF
ROC: Receiver operating characteristic
TOAST: Trial of org 10172 in acute stroke treatment
CV: Cross validation
CCVD: Cardio-cerebrovascular disease.

## Supplementary Materials

Supplementary 1 Supplement Figure S1: difference analysis of 22 immune cells infiltration separately in cardioembolic stroke (CE) obtained from patients with CE at each of the three time points ≤ 3 h (A), 5 h (B), and 24 h (C) following the stroke event compared to controls in GSE58294.

Supplementary 2 Supplementary Table S1: the characteristics of GEO datasets in this study.

Supplementary 3 Supplementary Table S2: clinical information of the stroke patients.
