# Supplementary material for: C1QC, VSIG4, and CFD as Potential Peripheral Blood Biomarkers in Atrial Fibrillation-Related Cardioembolic Stroke
Source: Oxid Med Cell Longev. 2023 Jan 5;2023:5199810. doi: 10.1155/2023/5199810 (PMC9837713; doi:10.1155/2023/5199810)
Supplement: Supplementary 1 — Supplement Figure S1: difference analysis of 22 immune cells infiltration separately in cardioembolic stroke (CE) obtained from patients with CE at each of the three time points ≤ 3 h (A), 5 h (B), and 24 h (C) following the stroke event compared to controls in GSE58294. [file 5199810.f1.doc]

| GEO serise | Platform | Samples | Organism | Country | Contributors |
| --- | --- | --- | --- | --- | --- |
| GSE58294 | GPL570 | 92 peripheral blood samples from 23 blood samples from patients with CE at three time points (<3 h, 5 h, 24 h) following the stroke event and 23 controls | Homo sapiens blood | USA | Stamova B et al., 2014 |
| GSE41177 | GPL570 | 19 left atrial tissue samples from 3 SR individuals and 16 AF patients | Homo sapiens | Taiwan, China | Yeh YH et al., 2013 |
| GSE14975 | GPL570 | 10 left atrial tissue samples from 5 SR individuals and 5 AF patients | Homo sapiens | Germany | Adam O B et al., 2010 |
| GSE115574 | GPL570 | 29 Left atrial tissue samples from 15 SR individuals and 14 AF patients | Homo sapiens | Turkey | Deniz GC et al., 2021 |
| GSE20129 | GPL10558 | 135 peripheral blood samples from 57 samples with atherosclerosis and 78 non-atherosclerosis controls | Homo peripheral blood | USA | Huang CC et al., 2012 |
| GSE Gene Expression Omnibus; CE: cardioembolic stroke; AF Atrial Fibrillation; SR Sinus Rhythm； | | | | | |

**Supplementary Table S1. Characteristics of GEO datasets in this study**
