# Supplementary figures and images for: C1QC, VSIG4, and CFD as Potential Peripheral Blood Biomarkers in Atrial Fibrillation-Related Cardioembolic Stroke
Source: Oxid Med Cell Longev. 2023 Jan 5;2023:5199810. doi: 10.1155/2023/5199810 (PMC9837713; doi:10.1155/2023/5199810)

A  
GSE58294 3h post-CE VS Control

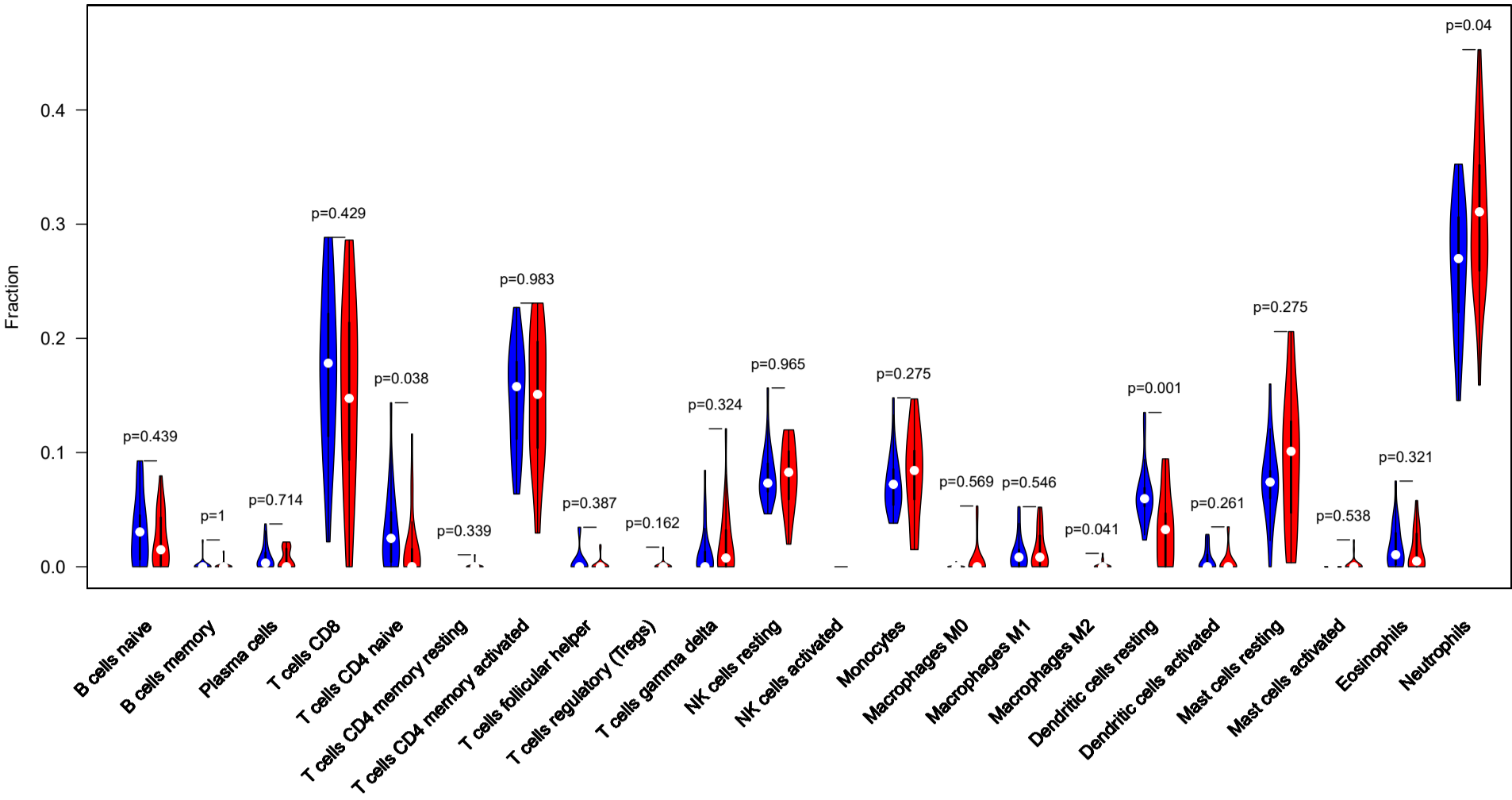

B  
GSE58294 5h post-CE VS Control

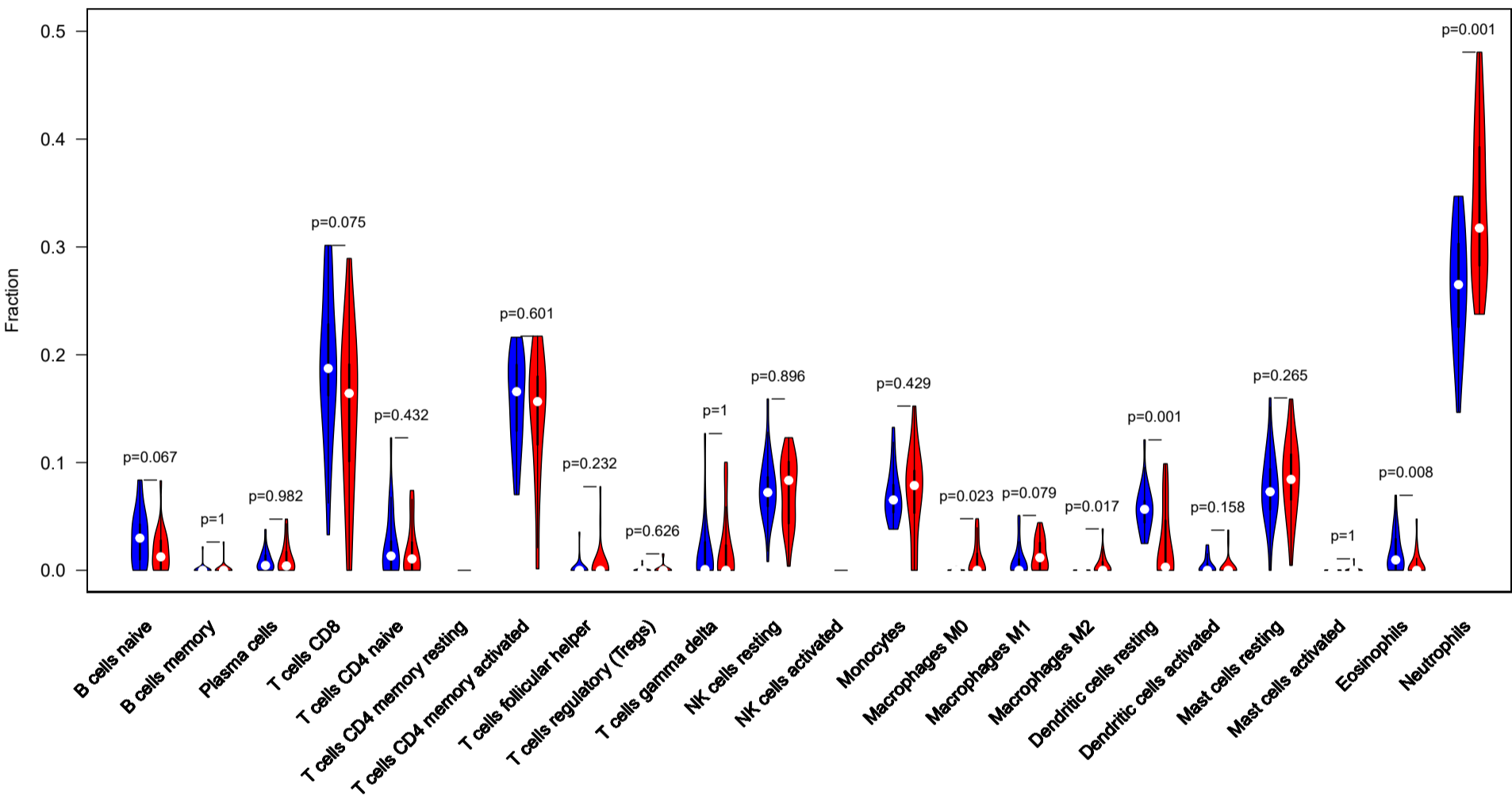

C  
GSE58294 24h post-CE VS Control

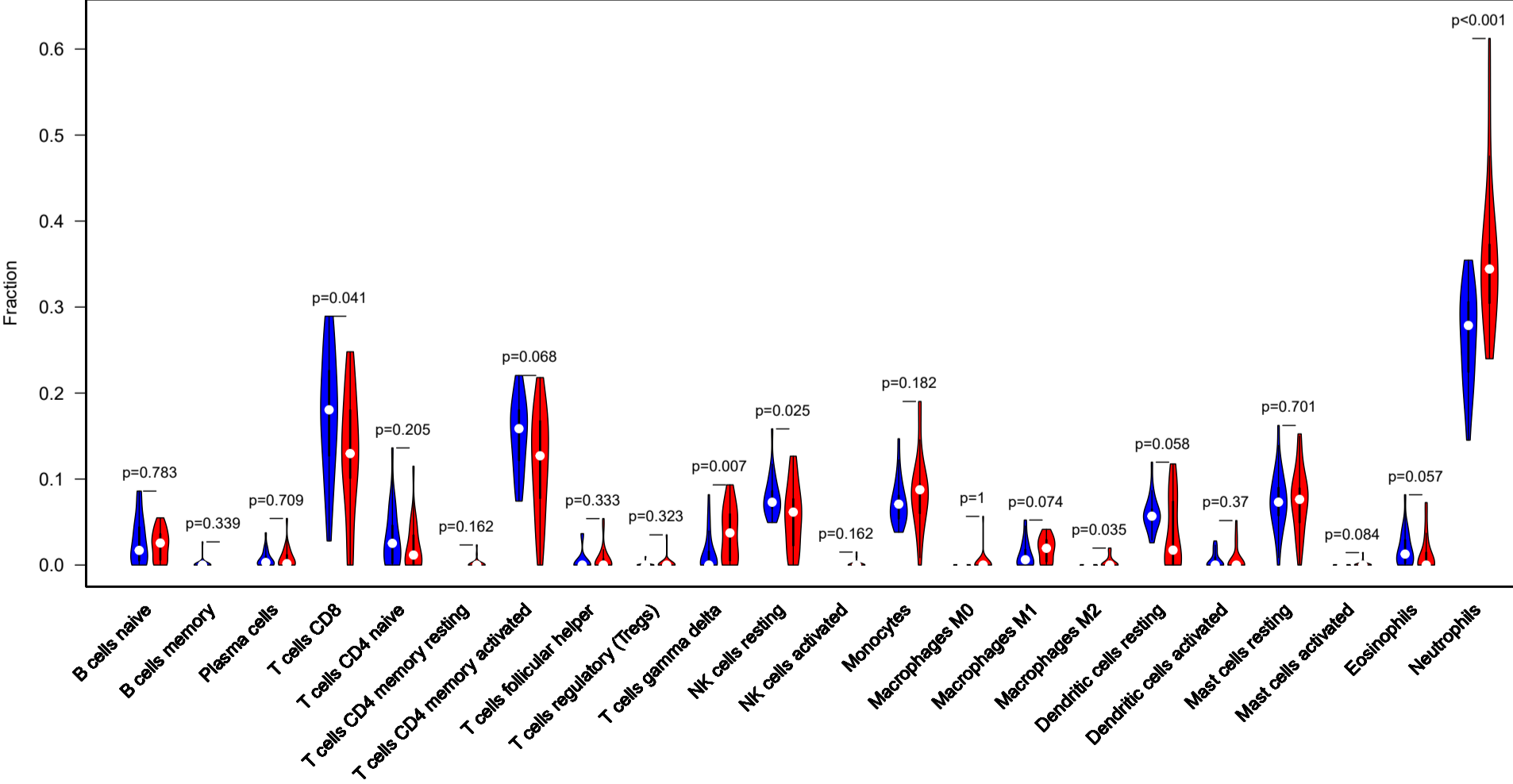

Supplement: Supplementary 2 — Supplementary Table S1: the characteristics of GEO datasets in this study. [file 5199810.f2.pdf]
