# Supplementary material for: C1QC, VSIG4, and CFD as Potential Peripheral Blood Biomarkers in Atrial Fibrillation-Related Cardioembolic Stroke
Source: Oxid Med Cell Longev. 2023 Jan 5;2023:5199810. doi: 10.1155/2023/5199810 (PMC9837713; doi:10.1155/2023/5199810)
Supplement: Supplementary 3 — Supplementary Table S2: clinical information of the stroke patients. [file 5199810.f3.pdf]

Supplementary Table S 2. Clinical information of the stroke patients

Stroke Samples ID	Sex	Age	TIA	Cardiogenic diseases	palpitation	pectoralgia	Asthma	syncope	hypertension	diabetes	Hyperlipidemia	Smoking
CE-1	male	79	NO	YES	YES	NO	YES	NO	YES	NO	NO	NO
CE-2	female	56	NO	YES	NO	NO	NO	NO	NO	NO	NO	YES
CE-3	female	40	NO	YES	YES	NO	NO	NO	NO	NO	NO	NO
CE-4	female	74	NO	YES	NO	NO	YES	YES	NO	NO	NO	NO
CE-5	female	85	NO	YES	NO	NO	YES	NO	NO	NO	NO	NO
CE-6	male	85	NO	YES	YES	NO	YES	NO	YES	NO	NO	NO
CE-7	male	59	NO	YES	NO	NO	NO	NO	YES	YES	NO	YES
CE-8	male	74	NO	NO	NO	NO	NO	NO	NO	NO	NO	NO
CE-9	female	75	NO	YES	NO	NO	YES	NO	NO	NO	NO	NO
CE-10	male	79	NO	YES	NO	NO	YES	NO	NO	YES	NO	NO
CE-11	female	74	YES	YES	NO	NO	NO	NO	YES	NO	NO	NO
CE-12	female	67	NO	NO	NO	NO	NO	NO	NO	NO	NO	NO
CE-13	male	64	NO	YES	NO	NO	NO	NO	YES	NO	NO	NO
CE-14	male	78	NO	YES	YES	NO	YES	NO	YES	NO	NO	NO
CE-15	male	73	NO	NO	NO	NO	NO	NO	NO	NO	NO	NO
CE-16	female	67	NO	YES	NO	NO	YES	NO	YES	NO	NO	YES
CE-17	female	64	NO	YES	NO	NO	NO	NO	YES	NO	NO	NO
CE-18	male	69	NO	YES	NO	NO	NO	NO	YES	YES	NO	NO
CE-19	female	60	NO	NO	NO	NO	YES	NO	YES	YES	NO	NO
CE-20	male	70	NO	YES	NO	NO	NO	NO	NO	NO	NO	NO
LAA-1	male	72	YES	NO	NO	NO	NO	NO	YES	YES	NO	YES
LAA-2	male	71	NO	YES	NO	NO	NO	NO	YES	NO	NO	YES
LAA-3	female	68	NO	NO	NO	NO	NO	NO	YES	NO	NO	NO
LAA-4	male	62	YES	NO	NO	NO	NO	NO	YES	NO	NO	YES
LAA-5	male	48	NO	NO	NO	NO	NO	NO	YES	NO	NO	YES
LAA-6	male	53	YES	NO	NO	NO	NO	NO	YES	NO	NO	YES
LAA-7	male	67	NO	NO	NO	NO	NO	NO	NO	NO	NO	NO
LAA-8	female	56	NO	NO	NO	NO	NO	NO	YES	NO	NO	YES
LAA-9	male	67	NO	NO	NO	NO	NO	NO	NO	NO	NO	NO
LAA-10	male	66	NO	NO	NO	NO	NO	NO	YES	NO	NO	NO
LAA-11	female	72	YES	NO	NO	NO	NO	NO	YES	YES	NO	NO
LAA-12	female	55	YES	NO	NO	NO	NO	NO	YES	NO	NO	NO
LAA-13	male	57	NO	NO	NO	NO	NO	NO	NO	YES	NO	NO
LAA-14	female	79	YES	NO	NO	NO	NO	NO	YES	YES	NO	YES
LAA-15	male	64	NO	YES	YES	NO	NO	NO	YES	NO	NO	NO
LAA-16	male	59	NO	NO	NO	NO	NO	NO	NO	NO	NO	NO
LAA-17	female	67	NO	NO	NO	NO	NO	NO	NO	NO	NO	YES
LAA-18	female	71	NO	YES	YES	NO	NO	NO	YES	YES	NO	NO
LAA-19	female	65	NO	YES	YES	NO	NO	NO	NO	NO	NO	NO
LAA-20	female	58	NO	NO	NO	NO	NO	NO	YES	NO	NO	NO
